# Pea Protein Nanoemulsion Effectively Stabilizes Vitamin D in Food Products: A Potential Supplementation during the COVID-19 Pandemic

**DOI:** 10.3390/nano11040887

**Published:** 2021-03-31

**Authors:** Yazan Akkam, Taha Rababah, Rui Costa, Ali Almajwal, Hao Feng, Juan E. Andrade Laborde, Mahmoud M. Abulmeaty, Suhail Razak

**Affiliations:** 1 Department of Medicinal Chemistry and Pharmacognosy, Faculty of Pharmacy, Yarmouk University, Irbid 21163, Jordan; 2 Department of Nutrition and Food Technology, Jordan University of Science and Technology, Irbid 22110, Jordan; 3 Instituto Politécnico de Coimbra, Escola Superior Agrária, Research Centre for Natural Resources, Environment and Society (CERNAS), Bencanta, 3045-601 Coimbra, Portugal; ruicosta@esac.pt; 4 Department of Community Health Sciences, College of Applied Medical Sciences, King Saud University, Riyadh 11433, Saudi Arabia; aalmajwal@ksu.edu.sa (A.A.); mabulmeaty@ksu.edu.sa (M.M.A.); smarazi@ksu.edu.sa (S.R.); 5 Department of Food Science and Human Nutrition, University of Illinois at Urbana-Champaign, Urbana, IL 61801, USA; haofeng@illinois.edu; 6 Food Science and Human Nutrition Department, University of Florida, Gainesville, FL 32611, USA; jandrade2@ufl.edu

**Keywords:** pea protein, COVID-19, vitamin D, nanoemulsion, sensory evaluation, UV stability, food products

## Abstract

Vitamin D deficiency is a global issue which has been exacerbated by the COVID-19 pandemic-related lockdowns. Fortification of food staples with vitamin D provides a solution to alleviate this problem. This research explored the use of pea protein nanoemulsion (PPN) to improve the stability of vitamin D in various food products. PPN was created using a pH-shifting and ultrasonication combined method. The physicochemical properties were studied, including particle size, foaming ability, water holding capacity, antioxidant activity, and total phenolic contents. The fortification of several food formulations (non-fat cow milk, canned orange juice, orange juice powder, banana milk, and infant formula) with vitamin D–PPN was investigated and compared to raw untreated pea protein (UPP) regarding their color, viscosity, moisture content, chemical composition, vitamin D stability, antioxidant activity, and morphology. Finally, a sensory evaluation (quantitative descriptive analysis, and consumer testing) was conducted. The results show that PPN with a size of 21.8 nm protected the vitamin D in all tested products. PPN may serve as a potential carrier and stabilizer of vitamin D in food products with minimum effects on the taste and color. Hence, PPN may serve as a green and safe method for food fortification during the COVID-19 pandemic.

## 1. Introduction

Vitamin D insufficiency and deficiency is a widespread global issue; it affects more than one billion children and adolescents all over the world [1,2]. Vitamin D as an essential micronutrient plays an important role in bone protection and metabolism [3]. Moreover, it also exhibits an important role in the innate and adaptive immune responses, and the prevention of neurodegenerative diseases, cancers, and cardiovascular diseases [3].

Coronavirus disease 2019 (COVID-2019)—a highly contagious virus—was confirmed as a pandemic on 12 March 2020, and has since spread worldwide [4]. COVID-19 is the leading cause of acute respiratory distress syndrome (ARDS) with a mortality rate of 3.7% [5]. Unfortunately, the only management is supportive (palliative) therapy [6]. Therefore, to prevent the contagion, government agencies around the world have recommended quarantine to reduce human-to-human contact via enforcing lockdown or curfew, which has subsequently led to less exposure to the sun, reinforcing vitamin D deficiencies. During the COVID-19 pandemic, vitamin D supplementation was proposed to reduce the risk of infection of coronavirus [1,7]. New evidence has shown that vitamin D activates cathelicidins and defensins which lower viral replication and down-regulate the cytokines; thus, decreasing the risk of respiratory failure and reducing the rate of infection [7].

Vitamin D (cholecalciferol) can be obtained from skin synthesis after sunlight exposure and ingestion via food or drink. Due to cultural and geographical reasons, sunlight exposure has been reduced among populations. Moreover, a large proportion of vitamin D in food is degraded due to food processing and inadequate storage conditions; such as the pH, salt, oxygen, light, and temperature [8]. Hence, sunlight is the only viable option to obtain vitamin D, and in most cases, supplements are required.

As a response to this pandemic, one strategy is the fortification of foods with readily bioaccessible vitamin D without the need for vitamin D supplementation. Pea protein has unique physicochemical characteristics that could be used to disperse fat-soluble compounds. Pea protein has been used in different food products such as in soybean, egg, or milk-derived products [9]. Furthermore, pea protein is lactose-free and safe for people with allergies or sensitivities to dairy, soybeans, and eggs [10]. Moreover, it has several advantages such as a high nutritional value, emulsification properties, relatively lost cost, and readily available [11]. The characteristics of pea protein have extensively been studied [12,13,14,15,16]. Hence, pea proteins have been utilized as rich bases in many food applications [17,18], and served as potential candidates for industrial products [19,20]. Unfortunately, pea protein’s low solubility is an obstacle that reduces its use as vehicles for the delivery of nutrients in foods [21]. It has been shown that the combination of ultrasonication and alkalinity improved the solubility of pea protein, reduced the particle size, enhanced the surface hydrophobicity, and disrupted the non-covalent interaction [21,22,23]. Furthermore, it has been proven that pea protein nanoemulsions (PPNs) protect vitamin D from UV light and heat [22]. Moreover, pea protein nanoaggregates synthesized using ultrasonication and alkalinity have been shown to be safe and successfully increased the bio-efficacy of vitamin D in rats [21]. Thus, the objective of this study was to explore the use of vitamin D–PPN fabricated using ultrasonication and alkalinity as a novel vehicle for food fortification strategy. In this regard, physicochemical properties, particle size, foaming ability, water holding capacity, antioxidant activity, total phenolic contents, and the protein composition of the PPN were studied. The fortification of several food formulations (non-fat cow milk, canned orange juice, orange juice powder, banana milk, and infant formula) with vitamin D–PPN was investigated and compared to raw untreated pea protein (UPP) regarding their color, viscosity, moisture content, chemical composition, vitamin D stability, antioxidant activity, and morphology. Finally, a sensory evaluation was conducted to touch upon quantitative and qualitative aspects of the taste and palatability of the formulation. This research is the first to evaluate pea protein nanoemulsion–vitamin D in food products and proved its efficiency using sensory evaluation.

## 2. Materials and Methods

Pea protein isolate (NUTRALYS^®^ S85F, 85% pea protein, dry basis) was provided by Roquette (Geneva, IL, USA). All reagents were purchased from Sigma-Aldrich (St. Louis, MO, USA) or Fisher Scientific (Pittsburgh, PA, USA). All food products were purchased from a local market (non-fat cow milk, canned orange juice, orange juice powder, banana milk, and infant formula).

### 2.1. pH-Shifting and Ultrasonication Treatment

Protein nano-aggregates using pH-shifting and ultrasonication treatment were prepared as described previously [22,23]. Raw pea protein (UPP) (3% w/v water) was treated with a different alkaline solution using 2 M NaOH (from pH 9–12) with and without 5 min sonication using a VC-750 ultrasonic processor at 20 kHz (Sonics & Materials, Inc., Newtown, CT, USA).

### 2.2. Production of Nanoemulsions (PPN) and PPN-Vitamin D

PPN was prepared as described previously [22]. Briefly, 1.0% (w/w) cholecalciferol/canola oil mixed with treated nano-pea protein aggregates/water solution (10 mg/mL) reaching a concentration of 20 µg vitamin D/mL. The mixture was then stirred for 5 min and sonicated for 5 min.

### 2.3. Particle Size and Zeta Potential Determination

The particle size experiments were conducted as described previously in triplicate [22,24]. Volume-weighted means diameters of the soluble proteins and nanoemulsions were measured using dynamic light scattering with a Zetasizer Nano S (model ZEN 1600, Malvern Instruments Ltd., Worcester, UK). Samples were diluted 500-fold with deionized water, and the measurement was conducted at 23 °C. The liquid viscosity set according to water was 0.933. The zeta potential for the smallest PPN was conducted as described previously [24]. The refractive indexes of the particle and dispersion medium were set at 1.52 and 1.33, respectively.

### 2.4. Color Measurement

The color readings of the samples (UPP, PPN, and food products) were obtained using a colorimeter (Color Tec-PCMTm Pittsford, New York, NY, USA) and recorded with the L*a*b* color system [25]. The L*a*b* color system consists of a luminance or lightness component (L*) and two chromatic components: the a* component for green (−a) to red (+a) and the b* component from blue (−b) to yellow (+b) colors. The colorimeter was calibrated using a standard white plate. Values of the white standard were L = 97.10, a = +0.13, b = +1.88, c = 1.88 and *h*^o^ = 86.1. Color measurements were averaged in triplicate.

### 2.5. Foaming and Water Holding Capacity

Foaming property was determined using the method of Alu’datt et al. [26] with slight modifications. UPP and PPN samples of 0.5 g were dissolved in 50 mL distilled water. The liquid was homogenized for 5 min at a high speed using a magnetic plate stirrer (VS.130SH, Scientific Co., LTD, Daejeon-Si, Korea) and the volume of the separated foam was taken.

The following equation was then utilized:

(1) $$\mathrm{Foaming}\;\mathrm{Capacity}\;=\;\frac{\mathrm{Vol}.\;\mathrm{after}\;\mathrm{homogenization}\;-\;\mathrm{Vol}.\;\mathrm{before}\;\mathrm{homogenization}}{\mathrm{Vol}.\;\mathrm{after}\;\mathrm{homogenization}}\times \;100\;$$

For water holding capacity determination, the method of Sathe and Salunkhe (1981) was adopted [26]. Five milliliters of distilled water were added to 0.5 g of UPP and PPN samples then mixed for 5 min. Thereafter, they were centrifuged for 30 min at 3500 rpm (Bench Top Centrifuge, NF 200, ANKARA, Turkey). The supernatant was discarded, and the tube was weighed. Water holding capacities were expressed as the gram of water retained per gram of sample, and was calculated using the following equation:

(2) $$\mathrm{Water}\;\mathrm{Holding}\;\mathrm{Capacity}\;=\;\frac{\;\mathrm{weight}\;\mathrm{of}\;\mathrm{the}\;\mathrm{sediment}\;-\mathrm{weight}\;\mathrm{of}\;\mathrm{the}\;\mathrm{dry}\;\mathrm{sample}}{\mathrm{weight}\;\mathrm{of}\;\mathrm{the}\;\mathrm{dry}\;\mathrm{sample}}\times \;100\;$$

### 2.6. Emulsion Capacity

The emulsion capacity was determined by the Beuchat method [27] (1977). Samples of UPP and PPN (0.1 g) were mixed with 5 mL distilled water for 2 min by a vortex. Afterwards, 0.5 mL canola oil were added, and the mixture was finally allowed to stand in a graduated cylinder. The separated water was measured after 1, 12, and 24 h.

### 2.7. Determination of Radical DPPH-Scavenging Activity (Antioxidant Activity).

The DPPH-scavenging activity was calculated as described previously [28]. A 500 μL sample of UPP or PPN (10 mg/mL water) was reacted with 0.2 mL of 1,1-diphenyl-2-picrylhydrazyl (DPPH) solution and then diluted to 4.0 mL using methanol. Samples were mixed thoroughly and allowed to stand in the dark for 30 min at room temperature. The absorbance was measured at 515 nm using a spectrophotometer (CELL, model CE 1020, Cecil Instruments, Cambridge, U.K.). Methanol was used as a blank. The radical-scavenging activity is defined as the percentage of inhibition:

(3) $$\mathrm{Inhibition}\left(\%\right)=\;\frac{\mathrm{Absorbance}\;\mathrm{of}\;\mathrm{blank}\;-\;\mathrm{Absorbance}\;\mathrm{of}\;\mathrm{sample}}{\mathrm{Absorbance}\;\mathrm{of}\;\mathrm{blank}}\times \;100\;$$

### 2.8. Total Phenolic Compounds

Determination of total phenolics was conducted according to the Folin−Ciocalteu method [29]. Fifty milligrams of UPP or PPN were vortexed with 25 mL of the extraction solvent (40 mL acetone: 40 mL methanol: 20 mL water: 0.1 mL formic acid (85%)). Then, samples were heated at 60 °C in a water bath for 1 h and cooled to room temperature before being homogenized for 30 s with a sonicator (Virtishear Tempest, The Virtis Co., New York, NY, USA). The homogenized sample was then filtered via Whatman filter paper.

The filtrates (200 μL) were mixed with 1.0 mL of Folin−Ciocalteu’s reagent and 1.0 mL of sodium carbonate (7.5%) before vortexing and incubating for 2 h. The absorbance at 726 nm was measured (Perkin-Elmer UV−vis spectrophotometer, Norwalk, CT, USA). The total phenolic content was expressed as chlorogenic acid equivalents (CAEs) in milligrams per gram of dry material according to the following equation:

(4) $$Total\;phenolics\;concentration\;\left(\frac{mg}{g}\right)=\left(\frac{A}{b}\right)\frac{SW+25}{SW}\;$$

where *A* is the absorbance at 726 nm, *SW* is the sample weight (g), and *b* is the slope of the standard curve of chlorogenic acid (50–400 µg/mL).

### 2.9. Separation Using SDS-PAGE

The SDS-PAGE was conducted as described previously [30]. The UPP and PPN samples were mixed with sample buffer in 1:1 ratio (for 5× sample buffer: 0.6 mL of 1 M Tris-HCl pH 6.8, 5 mL of 50% glycerol, 2 mL of 10% SDS, 0.5 mL of mercaptoethanol, 1 mL of 1% bromophenol blue, and 0.9 mL of H_2_O are mixed) and heated at 90 °C for three min.

Gradient PAGE: Mini-Protean TGX Precast Gels (BioRad, Hercules, CA, USA) were used.

### 2.10. Fortification of Food Products with Vitamin D–PPN

Each of the tested products was fortified with 2 µg vitamin D (100 µL of the stock) in the form of vitamin D (VD)–PPN per serving of 100 g product. A control containing the same amount of vitamin D and UPP was used.

### 2.11. Viscosity in Formulations

The viscosity analysis was conducted according to Pineda, 2007. A rotational cone-plate viscometer Brookfield CAP2000+ was used to measure the samples at a fixed temperature (25 °C). The viscosity was calculated based on the speed and the geometry of the probe.

### 2.12. Proximate Chemical Analysis

Standard AOAC (Association of Official Analytical Chemists) methods for moisture content (AOAC 925.09), total fat (AOAC 922.06), ash (AOAC 923.03), protein (AOAC 992.15), and carbohydrate (CHO) content of food formulation with and without UPP and PPN were determined according to the Association of Official Analytical Chemists (AOAC, 1984).

### 2.13. Protection Against UV-Light

Different sample formulations were suspended in special culture dishes similar to those published by Semo et al. [31]. Samples were exposed to UV light for 1, 3, 6, 12, and 24 h. Three types of samples were compared: two controls (positive and negative) and PPN. The positive control was the same formulation with vitamin D–PPN covered with a protective UV shield. The negative control was the formulation with vitamin D without PPN. Samples were taken at the specified times, and prepared and analyzed as described earlier.

### 2.14. Determination of Vitamin D Content in Food Formulations

A volume of 500 μL of each food product (ready or reconstituted) was mixed with 50 μL of formic acid (85%), vortexed for 6 min, and left to stand for 15 min at room temperature. Then, 250 μL of hexane was added, vortexed for 1 min, and then incubated for 10 min. Later, the supernatant (100 μL) was transferred to a large vial, dried under nitrogen, and then reconstituted by 200 μL of the mobile phase. Vitamin D3 (cholecalciferol) content in the food formulations was determined using the method of Semo et al. [31] on a Waters Chromatography system equipped with a Coulochem II system (electrochemical). The separation took place in a Zorbax C18 (4.6 × 150 mm) column using a mobile phase (1 acetonitrile: 4 methanol) at a flow rate of 1 mL/min. Detection was carried out at 265 nm with an electrochemical detector using several oxidation potentials (500, 700, 800 mV).

### 2.15. Sensory Evaluation

Quantitative descriptive analysis (QDA) was conducted as described previously [32]. The sensory characteristics were evaluated by nine panelists on a 9-point scale, with 9 = Extremely like, and 1 = Extremely dislike, and the recordings were analyzed using the general linear model (GLM) with SAS Version 8.2 software package (SAS 2002 Institute Inc., Cary, NC, USA) (SAS, 2002). Means were separated using Fisher’s Least Significant Difference (LSD) analysis at *p* ≤ 0.05.

Consumer testing was performed with a total of 75 participants ranging from 18–60 years old adults from various economic backgrounds. The participants were given written instructions and directed to taste at individual tables. Samples were coded with three random digit numbers and presented in a balanced order. Each participant was provided with sixteen plates containing 100 mL of each treatment.

To eliminate carry-over factors, the participants were also provided with green apple and plain biscuits for mouth cleansing between samples. The consumers were asked to record acceptability and intensity scores using the previously mentioned 9-point scale for each attribute.

### 2.16. Statistical Analysis

Results are reported as the mean and standard deviation based on the independent experiments. All experiments were conducted in triplicate with three independent experiments. The differences were analyzed using ANOVA with the JMP statistical package (JMP Institute Inc., Cary, NC, USA). Significant differences (*p* ≤ 0.05) between means were identified by Fisher’s least significant difference test.

## 3. Results and Discussion

### 3.1. Studying the Characteristics of PPN

#### 3.1.1. The Particle Size and Zeta Potential of PPN

**Figure 1:**
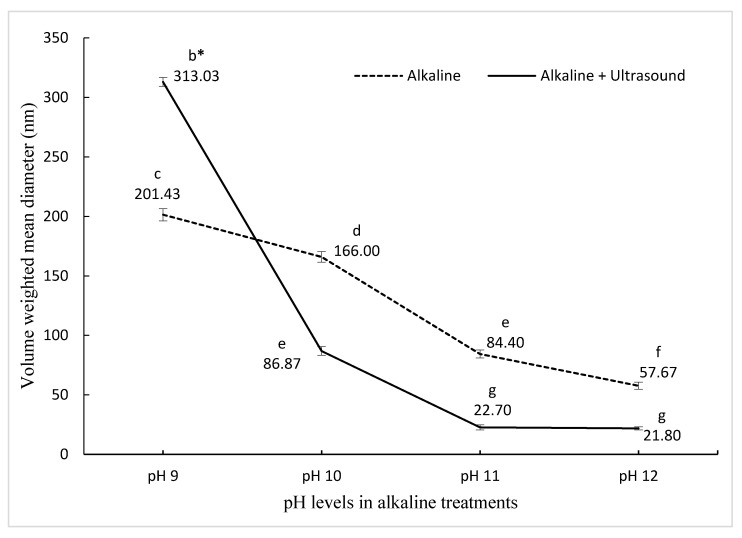
The effect of pH on pea nanoemulsion particle size synthesized using two different treatments. ^bcdefg^ Means (± standard deviation) with the same letter are not significantly different (*p* ≤ 0.05).

The initial particle size of PPN prepared with pea protein nano-aggregates produced by pH-shifting at pH 9 was 201.43 nm, which then decreased significantly reaching 57.67 nm for the PPN made with nano-aggregates prepared at pH 12. This represents a 71.36% reduction in the size with increasing pH (Figure 1). Increasing the pH promotes an acid–base reaction within amino acids, reducing the protein size, which leads to an increased contact area between the water and the protein, resulting in higher protein solubility [33,34]. A smaller protein size contributes to a better adsorption rate in the oil–water interface, refining the emulsifying ability [35].

In ultrasound and pH-shifting combined treatments, the initial particle size of PPN was 313.03 nm for pH shifting at pH 9, and by increasing the alkalinity, the size reduced to 21.8 nm at pH 12, which corresponds to a 93.05% reduction in the size of PPN (Figure 1). The size reduction and spherical shape of PPN were confirmed by Transmission electron microscope (Figure S1). Other studies have also reported that ultrasonication resulted in a decrease in protein particle size, and thus the size of emulsions [34,35,36,37,38,39]. Ultrasonication decreased the sizes of vegetable and animal protein aggregates by disturbing the hydrogen bonding, electrostatic, and hydrophobic interactions [35]. Sonication decreases the electrostatic barrier by decreasing the negative charge on the proteins and contributing to the foaming properties. Small size protein aggregates are a desired characteristic. It has been reported previously [22] that the combination of pH12 + ultrasound treatment led to a decrease in PPN particle sizes, which was three-fold smaller than pH12 treatment alone. However, O’Sullivan et al. [35] reported that when ultrasound treatment was combined with pH 2 and pH 10 treatments, ultrasonication had little to no effect on the protein particle size. In this study, the PPN with the smallest diameter (21.8 nm) was selected for further experiments. The zeta potential for the smallest size was 26.8 ± 0.7 mV. The results are in agreement with previous reports [24,40,41].

#### 3.1.2. Color Measurement

The potential effect of alkaline–ultrasonic treatment on pea protein on the solution color was tested. The color of UPP and PPN samples in water was measured and recorded using the L*a*b* color system (AOAC, 1984). The results are summarized in Table 1. The PPN solution was darker, greener, and more yellow than UPP. It has been stated that L* is directly proportional to the size of the nanoemulsion; the larger the diameter of the nanoparticle, the higher the luminosity value [42]. Therefore, PPN scattered less light than UPP.

#### 3.1.3. Foaming and Water Holding Capacity (WHC)

The property of proteins to form stable foams is important in the production of a variety of foods [43]. Therefore, the foaming ability was tested. Foam formation is controlled by the adsorption of the foaming agent at the air–water interface and its ability to rapidly decrease surface tension; many biomacromolecules exert surface liquid–liquid interfaces [43]. UPP possessed a foaming ability of 4.3%, while the ability increased in PPN, reaching 6.2% (Table 1).

The ultrasonic treatment induced the unfolding of the protein, which enhanced the adsorption at the air–water interface. Additionally, it led to the formation of visco-elastic films, enhancing the stability of the foam [44]. Besides, ultrasonic treatment decreased the protein particle size, which increased the solubility, leading to a better foaming property. Ultrasonication of pea protein isolates (PPIs) for 30 min has shown an improved foaming ability, 1.38-fold higher than untreated PPI [45].

WHC is defined as a physical property and is the ability of a food structure to prevent water from being released from the three-dimensional structure of the protein [46]. Water binding capacity is a limiting factor in protein food applications; in particular, it plays a major role in the formation of food texture [46]. In general, pea protein exhibits a low WHC value, less than 3% [47]. The PPN exhibited a significantly higher WHC compared to UPP; the WHV was 2.4% and 2.7% for the UPP and PPN, respectively (Table 1). This may be explained by the formation of smaller size particles, which leads to higher surface area and better interaction with water.

#### 3.1.4. Emulsion Properties

Emulsifying properties are useful functional characteristics that play an important role in the development of new sources of plant protein products for use in foods [48]. The results indicated that PPN is more effective on water incorporation in the emulsion than UPP (Table 2). Smaller particle size leads to a more stable emulsion [49]. Accordingly, the ultrasonication of pea protein confers a better emulsion property. Additionally, denatured proteins have their hydrophobic groups exposed and the unraveling of proteins is less necessary, leading to improved stability [49,50]. Moreover, the higher charge on the protein surface provides further stability to the emulsion, because the adsorbed proteins have electrostatic repulsion between them, preventing flocculation and coalescence [51].

#### 3.1.5. Total Phenolic Content and Antioxidant Activity.

In general, polyphenolic compounds exhibit antioxidant activities [52]. The total phenolic contents of the UPP and PPN were 77.6 and 88.1 mg/g, respectively (Table 3). In the antioxidant study, the PPN (29.6%) possessed a higher activity than the untreated pea protein (25.4%). The PPN may have provided better protection of the polyphenol compounds in the pea protein, which later leads to higher antioxidant activities [53]. Moreover, the treatment of pea protein might free more cysteine–sulfhydryl groups to interact with the oxidizing agents or to chelate transition metals [54]. It has been reported that the combination of pH-shifting and ultrasonication exposed the internal sulfhydryl groups via protein unfolding or cleaving the disulfide bond. However, the concentration of cysteine in pea protein is low (0.2% of total protein) [55].

#### 3.1.6. SDS-PAGE

**Figure 2:**
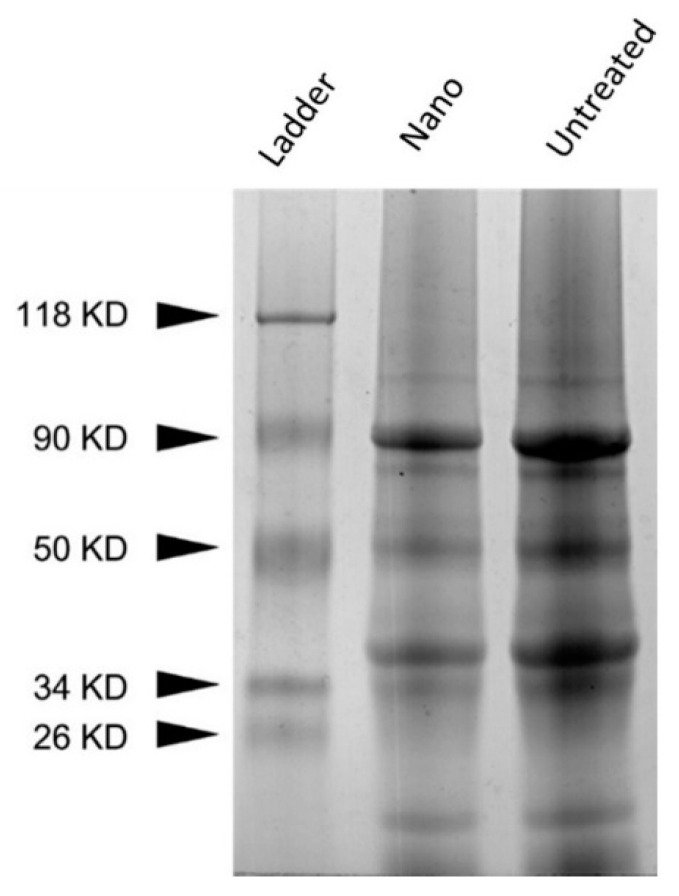
SDS-PAGE of UPP (untreated) and PPN (nano). Silver-stained gel.

Pea proteins are mainly composed of 70% globulins, vicilin, and legumin [56,57]. No significant differences in protein bands were observed between UPP and PPN (Figure 2). It has been stated previously that total pea proteins are separated into multiple components with molecular weight (MW) ranging from 104.8 kDa to 9.8 kDa, which mainly originated from vicilin and legumin [56]. According to Barać et al., 90 kDa is associated with lypoxigenase [30]. Furthermore, the bands 50 kDa, 38 kDa,34 kDa, and 18 kDa are related to proteolytic processing of the intact 50 kDa vicilin subunit. Additionally, legumin can be identified by bands at 40 kDa and 22 kDa. The treatment of pea protein to prepare PPN did not generate any aggregation or fibril formation. The alkaline pH-shifting and ultrasonication combined treatment also did not further degrade the pea proteins. The band above 100 kDa might be some polypeptide protein formed during the commercial processing of pea protein isolates [23].

### 3.2. Studying the Effect of Vitamin D/UPP and Vitamin D/PPN in Food Products

#### 3.2.1. Color, Viscosity, and Antioxidant Activity

The addition of vitamin D either in UPP or PPN did not change the color, viscosity, and antioxidant activity attributes of all the foods tested. Color is an important quality attribute in the food industry, which influences consumer’s choices and preferences. In this study, the effect of PPN and UPP were tested in commonly used food products, e.g., cow milk, infant formula, banana milk, fresh orange juice, and powdered orange juice.

Several studies have stated that increasing the viscosity of food may reduce food intake or suppress appetite [58]. Therefore, the addition of any supplement must not change the viscosity. The changes in color and viscosity were measured, and the results are summarized in Table 4. The results show that there were no significant changes in viscosity and color after the addition of VD/UPP or VD/PPN.

There was a slight change in the antioxidant activity between the control (product without any addition), PPN–food product, and UPP–food products. However, this change was not significantly different from the control (Table 4).

#### 3.2.2. Chemical Composition

The chemical composition of the tested food products was measured after the addition of VD/UPP or VD/PPN. As illustrated in Table 5, no statistically significant changes were observed after the addition.

#### 3.2.3. Stability in Food Products and Protection against UV Light

Nanoemulsion (oil-in-water) is considered an ideal model for bioactive compound encapsulation, which protects the encapsulated components from the environment [59]. Furthermore, the small size of nanoemulsion enhances the absorption of active agents (hydrophobic drug molecules) [60].

The ability of PPN to protect vitamin D in different food products from UV over a 24 h period was evaluated compared to UPP (Table 6). At time 0, the use of VD/UPP and VD/PPN significantly enhanced vitamin D content in all food additives. In all formulations, the nanoemulsion protected approximately more than 50% of the vitamin D over 24 h. While the addition of UPP protected around 15% of vitamin D. In all control samples, vitamin D degraded within 1 h of exposure.

Previously, it was reported that this type of nanoemulsion was capable of protecting vitamin D from UV radiation over 180 min with a recovery of 74% [22]. Several studies have evaluated the ability of protein-based nanoemulsions in protecting vitamin D [61,62,63,64]. However, most of the studies either did not expose vitamin D to UV light directly [61,63,64] or evaluated the stability of vitamin D for a duration of less than 9 h [62].

**Figure 3:**
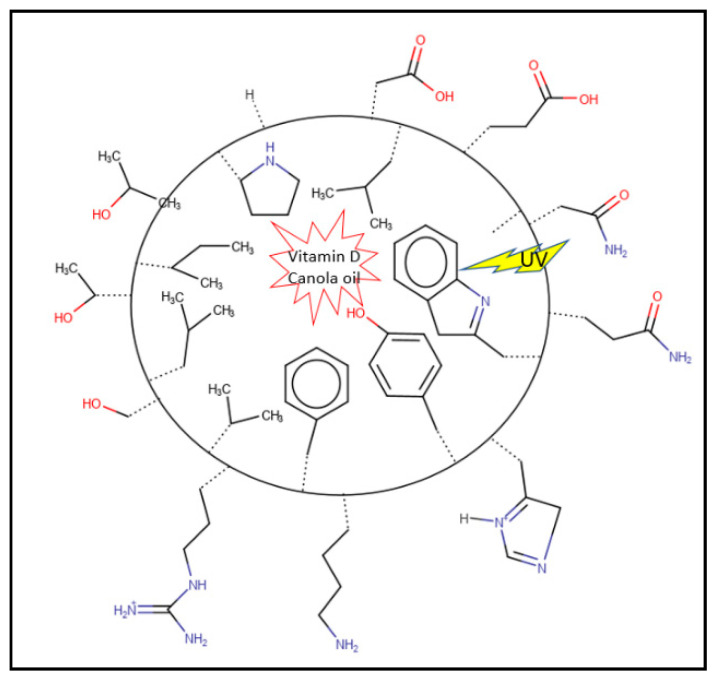
Diagram of a nanoemulsion droplet. The hydrophobic amino acid side chains are facing inside and interacting with vitamin D and canola oil, while the hydrophilic amino acids are exposing outside interactions with water. The aromatic amino acids and conjugated backbones are absorbing the UV and protecting the vitamin D.

Pea is rich in amino acids (except methionine and cysteine), especially branched and aromatic amino acids [11]. The treatment using alkaline pH-shifting and ultrasonication might expose the nonpolar amino acid residues, hence enhancing the hydrophobic interaction between pea protein and oil droplets and resulting in more stable emulsions (Figure 3).

The percentage of aromatic amino acids (phenylalanine and tyrosine) in pea’s protein is higher than in human muscle [55]. The aromatic side chains and double bonds in amino acids might absorb the UV light and hence protect the photochemical degradation of vitamin D (Figure 3).

Vitamin D can be altered or degraded by several factors other than UV, such as temperature and acidity [65]. Acidity is known to isomerize Vitamin D to isotachysterol, and this conversive can be facilitated by the air [66]. Hence, the presence of acidic vitamins such as ascorbic acid may catalyze isomerization. Moreover, it has been reported that vitamin D is degraded by oxidation and dehydration mechanism in an acidic medium [66]. The current project tested the stability of multiple variables, UV, and acidity. Orange juice and powders are acidic environments and contain a high level of ascorbic acid. The average pH of the commercial orange juice (ready and powder) is around 3 at room temperature [67]. The suggested mechanism is that the PPN was able to protect vitamin D from the chemical reactivity of the medium such as oxidizing agents and even air. There were no significant differences in the stability of vitamin D between acidic and neutral products (milk pH = 6.6).

#### 3.2.4. Sensory Evaluation

The results of quantitative descriptive analysis for different formulations were divided according to the overall impression, overall aroma, consistency, just-about-right consistency, overall color, and just-about-right color. The panelists evaluated the formulations without or with VD/UPP or VD/PPN. The results are tabulated in Table 7. No significant statistical changes were observed in all tested parameters. This might be related to the small amounts of UPP and PPN used in the study.

The population selected were asked to evaluate the samples according to four factors: overall impression, overall aroma, consistency, and overall color. No statistical changes in all parameters were observed. The results are shown in Table 8.

## 4. Conclusions

Protein nanoemulsion is shown to be an excellent candidate for vitamin D fortification. Pea protein’s nanoemulsion was successfully formulated using a basic medium (pH = 12) and ultrasonication, reaching approximately 22 nm in diameter. The physicochemical characteristics of PPN showed superiority over UPP, including a higher water holding capacity, higher foaming ability, better emulsion property, less color change, and higher antioxidant activities.

The use of PPN and vitamin D in food products (non-fat cow milk, canned orange juice, orange juice powder, banana milk, and infant formula) did not affect color, viscosity, chemical composition, or antioxidant activity, but successfully enhanced vitamin D content and protected it from UV degradation. The PPN also enhanced the stability of vitamin D in an acidic medium (isomerization and degradation) and protected it from air oxidation. The sensory evaluation using quantitative descriptive analysis and consumer testing confirmed that both the UPP and PPN containing vitamin D had a limited, if any, effect on the taste or preferences. Pea protein nanoemulsion may serve as a shuttle for vitamin D fortification during the coronavirus pandemic, reaching a large proportion of the population. Further research on long-term stability, the capacity of nanoemulsion to deliver a high concentration of vitamin D, and bioavailability in food products are suggested for future work.

## Figures and Tables

**Table 1:** Colorimetric study, foaming, and water holding capacity (WHC) of untreated pea protein (UPP) and pea protein nanoemulsion (PPN).

Samples	L*	a*	b*	Foaming%	WHC%
UPP	70.4 ± 5.1 ^a^	−0.61 ± 0.03 ^a^	24.5 ± 1.7 ^a^	4.3 ± 0.3 ^b^	2.4 ± 0.17 ^b^
PPN	60.5 ± 4.1 ^b^	−0.91 ± 0.07 ^b^	21.9 ± 1.5 ^b^	6.2 ± 0.44 ^a^	2.7 ± 0.19 ^a^

^ab^ Means with the same letter within the column are not significantly different (*p* ≤ 0.05).

**Table 2:** Emulsion capacity of both UPP and PPN.

Samples	Vol. of Water after 1 h (mL)	Vol. of Water after 12 h (mL)	Vol. of Water after 24 h (mL)
UPP	4.7 ± 0.37 ^a^	4.7 ± 0.29 ^a^	4.6 ± 0.43 ^a^
PPN	3.6 ± 0.27 ^b^	3.7 ± 0.31 ^b^	3.7 ± 0.25 ^b^

^ab^ Means within a column with the same letter are not significantly different (*p* ≤ 0.05).

**Table 3:** Total phenolic content and antioxidant activity of both UPP and PPN.

Treatment	Total Phenolics (mg/g)	Antioxidant Activity (%)
UPP	77.6 ± 6.4 ^b^	25.4 ± 1.7 ^b^
PPN	88.1 ± 5.2 ^a^	29.6 ± 2.1 ^a^

^ab^ Means within a column with the same letter are not significantly different (*p* ≤ 0.05).

**Table 4:** Analysis of color, viscosity, and antioxidant activity in formulations.

Treatment	L*	a*	b*	Viscosity (cP)	Antioxidant Activity (%)
Cow milk (Control)	81.52 ± 7.1 ^a^	−3.93 ± 0.3 ^a^	7.95 ± 0.4 ^a^	1.37 ± 0.08 ^a^	60.12 ± 5.3 ^a^
Cow milk vitamin D (VD)/UPP	81.48 ± 6.4 ^a^	−3.91 ± 0.2 ^a^	7.94 ± 0.9 ^a^	1.36 ± 0.14 ^a^	60.65 ± 4.8 ^a^
Cow milk VD/PPN	81.46 ± 8 ^a^	−3.88 ± 0.38 ^a^	7.97 ± 0.5 ^a^	1.38 ± 0.15 ^a^	60.73 ± 4.6 ^a^
Infant formula (Control)	78.34 ± 7 ^a^	−3.82 ± 0.07 ^a^	8.12 ± 0.4 ^a^	1.46 ± 0.16 ^a^	68.21 ± 6.1 ^a^
Infant formula VD/UPP	78.38 ± 6.2 ^a^	−3.80 ± 0.8 ^a^	8.08 ± 0.7 ^a^	1.45 ± 0.06 ^a^	68.45 ± 6.4 ^a^
Infant formula VD/PPN	78.26 ± 8.6 ^a^	−3.79 ± 0.6 ^a^	8.15 ± 0.5 ^a^	1.47 ± 0.01 ^a^	68.58 ± 6.7 ^a^
Orange juice (control)	67.82 ± 2 ^a^	3.74 ± 0.3 ^a^	53.2 ± 3.7^a^	2.14 ± 0.14 ^a^	24.17 ± 2.7 ^a^
Orange juice VD/UPP	67.84 ± 4.7 ^a^	3.73 ± 0.25 ^a^	53.1 ± 4.2 ^a^	2.14 ± 0.19 ^a^	24.25 ± 2.8 ^a^
Orange juice VD/PPN	67.81 ± 2.7 ^a^	3.71 ± 0.33 ^a^	53.2 ± 5.3 ^a^	2.16 ± 0.2 ^a^	24.31 ± 2.2 ^a^
Banana milk (Control)	73.54 ± 5 ^a^	−3.56 ± 0.42 ^a^	9.32 ± 0.6 ^a^	1.59 ± 0.2 ^a^	54.13 ± 4.8 ^a^
Banana milk VD/UPP	73.52 ± 1.8 ^a^	−3.55 ± 0.17 ^a^	9.35 ± 0.8 ^a^	1.58 ± 0.17 ^a^	54.56 ± 4.0 ^a^
Banana milk VD/PPN	73.51 ± 3.6 ^a^	−3.52 ± 0.33 ^a^	9.36 ± 0.6 ^a^	1.61 ± 0.04 ^a^	54.69 ± 5.1 ^a^
Orange juice powder (Control)	62.61 ± 6.8 ^a^	6.42 ± 0.6 ^a^	55.7 ± 6.1 ^a^	NA	16.34 ± 1.5 ^a^
Orange juice powder VD/UPP	62.27 ± 1.9 ^a^	6.46 ± 0.3 ^a^	55.6 ± 5.5 ^a^	NA	16.38 ± 1.4 ^a^
Orange juice powder VD/PPN	62.55 ± 5 ^a^	6.47 ± 0.18 ^a^	55.6 ± 2.2 ^a^	NA	16.42 ± 1.4 ^a^

NA: not applicable. ^a^ Means within a column with the same letter are not significantly different (*p* ≤ 0.05).

**Table 5:** The chemical composition of formulations after the addition of VD/UPP and VD/PPN.

Treatment	Moisture	Protein	Carbohydrates	Fat	Ash
Cow milk (Control)	86.81 ± 1.7 ^a^	3.28 ± 0.1 ^a^	4.38 ± 0.26 ^a^	3.52 ± 0.28 ^a^	0.64 ± 0.02 ^a^
Cow milk VD/UPP	86.80 ± 6.5 ^a^	3.28 ± 0.15 ^a^	4.38 ± 0.3 ^a^	3.52 ± 0.24 ^a^	0.64 ± 0.05 ^a^
Cow milk VD/PPN	86.80 ± 2.8 ^a^	3.29 ± 0.12	4.38 ± 0.17 ^a^	3.52 ± 0.14 ^a^	0.64 ± 0.03 ^a^
Infant formula (Control)	85.29 ± 3.4 ^a^	2.30 ± 0.18 ^a^	9.20 ± 0.4 ^a^	4.50 ± 0.3 ^a^	0.71 ± 0.05 ^a^
Infant formula VD/UPP	85.29 ± 7.5 ^a^	2.28 ± 0.09 ^a^	9.20 ± 0.5 ^a^	4.50 ± 0.2 ^a^	0.71 ± 0.03 ^a^
Infant formula VD/PPN	85.28 ± 4.6 ^a^	2.30 ± 0.14 ^a^	9.20 ± 0.4 ^a^	4.50 ± 0.27 ^a^	0.71 ± 0.06 ^a^
Orange juice (control)	87.33 ± 7 ^a^	0.62 ± 0.02 ^a^	9.64 ± 0.32 ^a^	NA	0.45 ± 0.03 ^a^
Orange juice VD/UPP	87.34 ± 5 ^a^	0.62 ± 0.018 ^a^	9.63 ± 0.2 ^a^	NA	0.45 ± 0.02 ^a^
Orange juice VD/PPN	87.35 ± 6.5 ^a^	0.62 ± 0.03 ^a^	9.64 ± 0.1 ^a^	NA	0.45 ± 0.03 ^a^
Banana milk (Control)	83.57 ± 4.3 ^a^	4.21 ± 0.12 ^a^	11.15 ± 0.2 ^a^	0.25 ± 0.02 ^a^	0.82 ± 0.05 ^a^
Banana milk VD/UPP	83.57 ± 5.2 ^a^	4.22 ± 0.25 ^a^	11.16 ± 0.6 ^a^	0.25 ± 0.01 ^a^	0.81 ± 0.05 ^a^
Banana milk VD/PPN	83.56 ± 8.7 ^a^	4.22 ± 0.3 ^a^	11.15 ± 0.3 ^a^	0.26 ± 0.02 ^a^	0.83 ± 0.04 ^a^
Orange juice powder (Control)	9.65 ± 0.9 ^a^	4.34 ± 0.3 ^a^	73.48 ± 5.1 ^a^	NA	4.15 ± 0.2 ^a^
Orange juice powder VD/UPP	9.65 ± 0.5 ^a^	4.33 ± 0.27 ^a^	73.48 ± 2.8	NA	4.16 ± 0.25 ^a^
Orange juice powder VD/PPN	9.65 ± 0.8 ^a^	4.34 ± 0.1 ^a^	73.47 ± 5.8 ^a^	NA	4.16 ± 0.14 ^a^

NA: not applicable. ^a^ Means within a column with the same letter are not significantly different (*p* ≤ 0.05).

**Table 6:** Vitamin D content (µg/100 mL) in all food products over 24 h.

Treatment	UV Exposure Time (h)					
	0	1	3	6	12	14
Cow milk (Control)	0.005 ± 0.001 ^a^	0.005 ± 0.001 ^a^	Trace	Trace	0	0
Cow milk VD/UPP	0.861 ± 0.06 ^a^	0.642 ± 0.04 ^b^	0.534 ± 0.06 ^c^	0.432 ± 0.08 ^d^	0.263 ± 0.04 ^e^	0.145 ± 0.09 ^f^
Cow milk VD/PPN	0.861 ± 0.05 ^a^	0.851 ± 0.08 ^a^	0.842 ± 0.01 ^a^	0.752 ± 0.02 ^b^	0.641 ± 0.06 ^c^	0.568 ± 0.06 ^d^
Infant formula (Control)	0.015 ± 0.001 ^a^	0.013 ± 0.001 ^a^	0.007 ± 0.001 ^b^	Trace	Trace	0
Infant formula VD/UPP	0.875 ± 0.06 ^a^	0.672 ± 0.09 ^b^	0.563 ± 0.01 ^c^	0.427 ± 0.04 ^d^	0.275 ± 0.01^e^	0.164 ± 0.03 ^f^
Infant formula VD/PPN	0.895 ± 0.09 ^a^	0.887 ± 0.08 ^a^	0.874 ± 0.04 ^a^	0.768 ± 0.05 ^b^	0.621 ± 0.01 ^c^	0.537 ± 0.07 ^d^
Orange juice (control)	Trace	0	0	0	0	0
Orange juice VD/UPP	0.860 ± 0.04 ^a^	0.624 ± 0.07 ^b^	0.486 ± 0.01 ^c^	0.379 ± 0.02 ^d^	0.253 ± 0.06 ^e^	0.128 ± 0.08 ^f^
Orange juice VD/PPN	0.860 ± 0.08 ^a^	0.848 ± 0.04 ^a^	0.834 ± 0.06 ^b^	0.741 ± 0.03 ^c^	0.592 ± 0.04 ^d^	0.511 ± 0.08 ^e^
Banana milk (Control)	0.005 ± 0.001 ^a^	0.005 ± 0.001 ^a^	Trace	0	0	0
Banana milk VD/UPP	0.861 ± 0.07 ^a^	0.611 ± 0.01 ^b^	0.527 ± 0.02 ^c^	0.417 ± 0.05 ^d^	0.264 ± 0.06 ^e^	0.143 ± 0.09 ^f^
Banana milk VD/PPN	0.861 ± 0.03 ^a^	0.851 ± 0.05 ^a^	0.845 ± 0.03 ^a^	0.732 ± 0.03 ^b^	0.614 ± 0.04 ^c^	0.527 ± 0.06 ^d^
Orange juice powder (Control)	Trace	0	0	0	0	0
Orange juice powder VD/UPP	0.862 ± 0.03 ^a^	0.642 ± 0.04 ^b^	0.489 ± 0.04 ^c^	0.384 ± 0.03 ^d^	0.261 ± 0.04 ^e^	0.138 ± 0.07 ^f^
Orange juice powder VD/PPN	0.862 ± 0.04 ^a^	0.851 ± 0.05 ^a^	8.42 ± 0.0.4 ^b^	0.725 ± 0.01 ^c^	0.584 ± 0.02 ^d^	0.514 ± 0.03 ^e^

^abcdefg^ Means within the row with the same letter are not significantly different (*p* ≤ 0.05).

**Table 7:** Quantitative descriptive analysis of various food products after vitamin D fortification using UPP and PPN.

Treatment	Overall Impression	Overall Aroma	Consistency	Consistency Just about Right	Overall Color	Color Just about Right
Cow milk (Control)	8.4 ± 0.6 ^a^	7.9 ± 0.5 ^a^	8.5 ± 0.1 ^a^	4.2 ± 0.1 ^a^	8.4 ± 0.3 ^a^	4.3 ± 0.2 ^a^
Cow milk VD/UPP	8.3 ± 0.5 ^a^	7.9 ± 0.6 ^a^	8.5 ± 0.5 ^a^	4.2 ± 0.2 ^a^	8.4 ± 0.2 ^a^	4.3 ± 0.2 ^a^
Cow milk VD/PPN	8.3 ± 0.3 ^a^	7.8 ± 0.6 ^a^	8.6 ± 0.4 ^a^	4.3 ± 0.3 ^a^	8.3 ± 0.4 ^a^	4.2 ± 0.2 ^a^
Infant formula (Control)	8.6 ± 0.2 ^a^	8.1 ± 0.5 ^a^	8.3 ± 0.2 ^a^	4.4 ± 0.1 ^a^	8.4 ± 0.6 ^a^	4.5 ± 0.3 ^a^
Infant formula VD/UPP	8.6 ± 0.5 ^a^	8.1 ± 0.3 ^a^	8.3 ± 0.6 ^a^	4.4 ± 0.2 ^a^	8.4 ± 0.7 ^a^	4.5 ± 0.3 ^a^
Infant formula VD/PPN	8.5 ± 0.3 ^a^	8.1 ± 0.6 ^a^	8.3 ± 0.2 ^a^	4.3 ± 0.3 ^a^	8.4 ± 0.5 ^a^	4.5 ± 0.14 ^a^
Orange juice (control)	8.2 ± 0.4 ^a^	8.4 ± 0.3 ^a^	8.1 ± 0.3 ^a^	4.5 ± 0.1 ^a^	8.5 ± 0.3 ^a^	4.3 ± 0.2 ^a^
Orange juice VD/UPP	8.3 ± 0.6 ^a^	8.4 ± 0.2 ^a^	8.1 ± 0.5 ^a^	4.5 ± 0.3 ^a^	8.5 ± 0.4 ^a^	4.3 ± 0.2 ^a^
Orange juice VD/PPN	8.2 ± 0.6 ^a^	8.4 ± 0.4 ^a^	8.3 ± 0.3 ^a^	4.6 ± 0.17 ^a^	8.4 ± 0.6 ^a^	4.2 ± 0.3 ^a^
Banana milk (Control)	8.5 ± 0.5 ^a^	8.6 ± 0.5 ^a^	8.4 ± 0.2 ^a^	4.6 ± 0.2 ^a^	8.5 ± 0.3 ^a^	4.4 ± 0.4 ^a^
Banana milk VD/UPP	8.5 ± 0.3 ^a^	8.6 ± 0.5 ^a^	8.4 ± 0.5 ^a^	4.6 ± 0.2 ^a^	8.5 ± 0.4 ^a^	4.4 ± 0.4 ^a^
Banana milk VD/PPN	8.5 ± 0.4 ^a^	8.5 ± 0.4 ^a^	8.4 ± 0.2 ^a^	4.5 ± 0.3 ^a^	8.4 ± 0.5 ^a^	4.2 ± 0.3 ^a^
Orange juice powder (Control)	8.3 ± 0.6 ^a^	8.1 ± 0.2 ^a^	8.2 ± 0.5 ^a^	4.3 ± 0.2 ^a^	8.2 ± 0.1 ^a^	4.2 ± 0.2 ^a^
Orange juice powder VD/UPP	8.3 ± 0.4 ^a^	8.1 ± 0.2 ^a^	8.2 ± 0.3 ^a^	4.3 ± 0.1 ^a^	8.2 ± 0.5 ^a^	4.2 ± 0.3 ^a^
Orange juice powder VD/PPN	8.3 ± 0.2 ^a^	8.1 ± 0.6 ^a^	8.1 ± 0.3 ^a^	4.3 ± 0.15 ^a^	8.2 ± 0.4 ^a^	4.2 ± 0.2 ^a^

^a^ Means within the column with the same letter are not significantly different (*p* ≤ 0.05).

**Table 8:** Consumer testing of various food products after Vitamin D fortification using UPP and PPN.

Treatment	Overall Impression	Overall Aroma	Consistency	Overall Color
Cow milk (Control)	8.2 ± 0.6 ^a^	7.8 ± 0.4 ^a^	8.3 ± 0.4 ^a^	8.2 ± 0.5 ^a^
Cow milk VD/UPP	8.1 ± 0.2 ^a^	7.7 ± 0.5 ^a^	8.3 ± 0.5 ^a^	8.1 ± 0.6 ^a^
Cow milk VD/PPN	8.1 ± 0.4 ^a^	7.6 ± 0.6 ^a^	8.1 ± 0.6 ^a^	8.1 ± 0.4 ^a^
Infant formula (Control)	8.4 ± 0.7 ^a^	8.3 ± 0.4 ^a^	8.2 ± 0.4 ^a^	8.2 ± 0.3 ^a^
Infant formula VD/UPP	8.2 ± 0.3 ^a^	8.3 ± 0.3 ^a^	8.1 ± 0.5 ^a^	8.1 ± 0.2 ^a^
Infant formula VD/PPN	8.2 ± 0.5 ^a^	8.1 ± 0.4 ^a^	8.3 ± 0.3 ^a^	8.1 ± 0.6 ^a^
Orange juice (control)	8.4 ± 0.5 ^a^	8.2 ± 0.5 ^a^	8.1 ± 0.2 ^a^	8.3 ± 0.5 ^a^
Orange juice VD/UPP	8.3 ± 0.6 ^a^	8.1 ± 0.3 ^a^	8.2 ± 0.6 ^a^	8.1 ± 0.4 ^a^
Orange juice VD/PPN	8.4 ± 0.2 ^a^	8.3 ± 0.6 ^a^	8.3 ± 0.4 ^a^	8.2 ± 0.4 ^a^
Banana milk (Control)	8.3 ± 0.2 ^a^	8.2 ± 0.6 ^a^	8.2 ± 0.6 ^a^	8.1 ± 0.4 ^a^
Banana milk VD/UPP	8.1 ± 0.4 ^a^	8.4 ± 0.4^a^	8.1 ± 0.3 ^a^	8.3 ± 0.5 ^a^
Banana milk VD/PPN	8.2 ± 0.3 ^a^	8.2 ± 0.2 ^a^	8.3 ± 0.1 ^a^	8.1 ± 0.2 ^a^
Orange juice powder (Control)	8.2 ± 0.1 ^a^	8.2 ± 0.1 ^a^	8.2 ± 0.4 ^a^	8.3 ± 0.2 ^a^
Orange juice powder VD/UPP	8.2 ± 0.2 ^a^	8.1 ± 0.5 ^a^	8.1 ± 0.3 ^a^	8.2 ± 0.1 ^a^
Orange juice powder VD/PPN	8.3 ± 0.3 ^a^	8.1 ± 0.6 ^a^	8.3 ± 0.3 ^a^	8.4 ± 0.1 ^a^

^a^ Means within the column with the same letter are not significantly different (*p* ≤ 0.05).

## Data Availability

Data presented in this study are available by request from the corresponding author.

## Supplementary Materials

The following are available online at https://www.mdpi.com/article/10.3390/nano11040887/s1, Figure S1 Transmission electron microscope images for pea proteins nanoemulsion.
