# Supplementary material for: Pea Protein Nanoemulsion Effectively Stabilizes Vitamin D in Food Products: A Potential Supplementation during the COVID-19 Pandemic
Source: Nanomaterials (Basel). 2021 Mar 31;11(4):887. doi: 10.3390/nano11040887 (PMC8065392; doi:10.3390/nano11040887)
Supplement: Supplementary file 1 [file nanomaterials-11-00887-s001.pdf]

nanomaterials

MDPI

Article

Pea Protein Nanoemulsion Effectively Stabilizes Vitamin D in Food Products: A Potential Supplementation During the COVID-19 Pandemic

Yazan Akkam [1,*], Taha Rababah [2,*], Rui Costa [3], Ali Almajwal [4], Hao Feng [5], Juan E. Andrade Laborde [6], Mahmoud M. Abulmeaty [4] and Suhail Razak [4]

1 Department of Medicinal Chemistry and Pharmacognosy, Faculty of Pharmacy, Yarmouk University, Irbid 21163, Jordan; yazan.a@yu.edu.jo

2 Department of Nutrition and Food Technology, Jordan University of Science and Technology, Irbid 22110, Jordan; trababah@just.edu.jo

3 Instituto Politécnico de Coimbra, Escola Superior Agrária, Research Centre for Natural Resources, Environment and Society (CERNAS), Bencanta, 3045-601 Coimbra, Portugal; ruicosta@esac.pt

4 Department of Community Health Sciences, College of Applied Medical Sciences, King Saud University, Riyadh 11433, Saudi Arabia; aalmajwal@ ksu.edu.sa (A.A.); mabulmeaty@ ksu.edu.sa (M.M.A.); smarazi@ksu.edu.sa (S.R.)

5 Department of Food Science and Human Nutrition, University of Illinois at Urbana-Champaign, Urbana, IL 61801, USA; haofeng@illinois.edu

6 Food Science and Human Nutrition Department, University of Florida, Gainesville, FL 32611, USA; jandrade2@ufl.edu

* Correspondence: yazan.a@yu.edu.jo (Y.A.); trababah@just.edu.jo (T.R.)

The transmission electron microscopy study of the smallest PPN was conducted using an FEI Titan Microscope (FEI company, Hillsboro, OR, USA) as described previously [1]. A drop of PPN mixed with a drop of sodium phosphotungstate (2% w/v) on TEM grids coated with carbon films. Then the sample was dried at room temperature. The results verified the real dimension and distribution of the PPN.

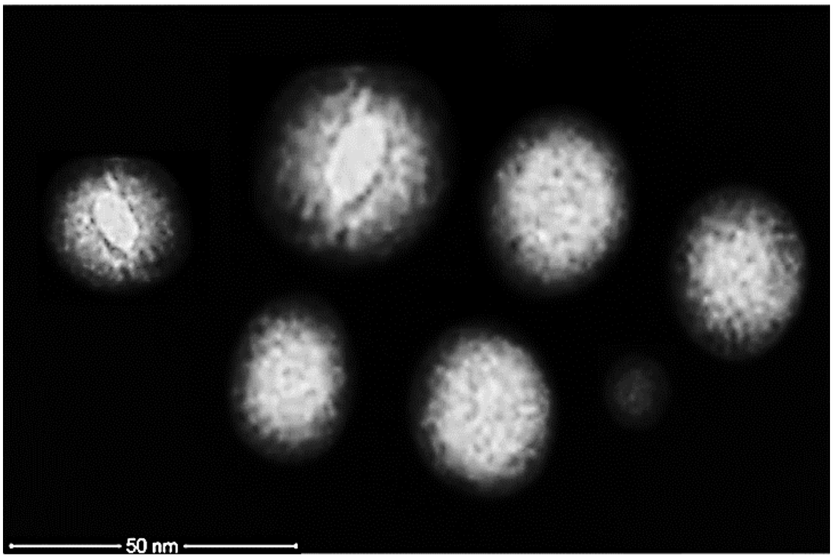

Figure S1. Transmission electron microscope images for pea proteins nanoemulsion

References

1. Walia, N.; Chen, L. Pea protein based vitamin D nanoemulsions: Fabrication, stability and in vitro study using Caco-2 cells. *Food Chemistry*, **2020**, *305*, 125475–125483.
